# Profiles of mental health indicators among Ukrainian people recently bereaved in the context of the full-scale Russian invasion

**DOI:** 10.1371/journal.pone.0358123

**Published:** 2026-11-03

**Authors:** Justina Pociūnaitė-Ott, Paul A. Boelen, Iryna Frankova, Iryna Norkina, Maria Luisa F. Rispa Hoyos, Lonneke I.M. Lenferink

**Affiliations:** 1 Department of Psychology, Health & Technology, Faculty of Behavioural Management and Social Sciences, University of Twente, Enschede, The Netherlands; 2 Department of Health Psychology, Lithuanian University of Health Sciences, Kaunas, Lithuania; 3 Psychology Institute, Mykolas Romeris University, Vilnius, Lithuania; 4 Department of Clinical Psychology, Faculty of Social and Behavioural Sciences, Utrecht University, Utrecht, The Netherlands; 5 ARQ National Psychotrauma Centre, Diemen, The Netherlands; 6 Department of Clinical, Neuro- and Developmental Psychology, WHO Collaborating Center for Research and Dissemination of Psychological Interventions, Amsterdam Public Health Institute, Vrije Universiteit Amsterdam, Amsterdam, The Netherlands; 7 Department of Medical Psychology, Psychosomatic Medicine and Psychotherapy, Bogomolets National Medical University, Kyiv, Ukraine; 8 Department of Clinical Psychology and Experimental Psychopathology, Faculty of Behavioral and Social Sciences, University of Groningen, Groningen, The Netherlands; Fakultet za pravne i poslovne studije dr Lazar Vrkatic, SERBIA

## Abstract

Bereavement research has frequently focused on psychopathology, while comparatively less attention has been given to well-being as a component of mental health. This study is the first to examine mental health profiles based on ICD-11 Prolonged Grief (PG) symptoms, posttraumatic stress (PTS) symptoms, and well-being in a sample of Ukrainians bereaved during the full-scale Russian invasion. Participants were 290 adults (*M*age = 34.3, *SD* = 9.1 years), who had lost a loved one on average 5.3 months earlier. Over a third (35%) had lost a partner or child, due to illness (43%) or war (40%). The majority of participants (55%) scored above the threshold of clinically relevant PG symptoms. Latent profile analyses identified three distinct mental health profiles: (1) low PG symptoms/low PTS symptoms/high well-being (13%), (2) moderate PG symptoms/low PTS symptoms/moderate well-being (57%), (3) high PG symptoms/low PTS symptoms/low well-being (30%). People evidencing the third profile were more likely to have experienced the loss of a close person (partner or child) and to have been forcibly displaced compared to people in other profiles. Notably, the distribution of these profiles differs from previous studies, where profiles marked by low symptoms and high well-being were typically most common. Moreover, PG and well-being appeared to lie on opposite ends of a single continuum rather than functioning as distinct dimensions, providing limited support for the dual-continua model. This pattern suggests that Ukrainians may be facing unique challenges due to ongoing war-related stressors. Identifying those most in need of support and highlighting potential intervention targets are particularly important in under-resourced populations where access to care is limited.

## Introduction

Psychological reactions to the loss of a loved one may vary from person to person. For the majority of people, the intense feelings of grief that follow the loss decrease within the first year after loss [1]. Some, however, develop psychopathology after loss, such as prolonged grief disorder (PGD) and/or posttraumatic stress disorder (PTSD), particularly after a traumatic loss, i.e., the loss that occurs under potentially traumatic circumstances [2–4]. PGD is characterized by separation distress, including persistent longing for the deceased and preoccupation with the deceased. PTSD is marked by re-experiencing symptoms after exposure to a traumatic event, such as nightmares and flashbacks. PGD shares some characteristics with PTSD (e.g., avoidance), yet factor, latent class, latent trajectory, and network analytic studies show that the two are related, but distinct [4–6]. This distinction highlights the importance of examining prolonged grief (PG) and posttraumatic stress (PTS) symptoms simultaneously to better understand bereavement-related mental health.

Bereavement research has often focused on negative emotional outcomes. However, psychopathology and well-being are two distinct yet related dimensions of mental health, as postulated by the dual-continua model [7,8]. The absence of psychopathology does not imply the presence of well-being, and the presence of well-being does not imply the absence of psychopathology. Therefore, focusing solely on the psychopathology would provide an overly one-sided picture of mental health. Recent research has shown that people who experienced a loss during the COVID-19 pandemic may simultaneously exhibit both PGD and well-being [9]. After a traumatic loss, a bereaved person with PGD does not necessarily experience below average well-being levels and vice versa [10]. Relatively little attention has been given to mental health after bereavement, particularly in terms of both psychopathology and well-being as coexisting dimensions, and when the loss occurs during an international armed conflict.

The Russian full-scale invasion of Ukraine on February 24, 2022, precipitated the largest conventional international armed conflict in Europe since the World War II. By 2025, Russia occupied Crimea and parts of Donetsk, Kharkiv, Kherson, Luhansk, Mykolayiv, and Zaporizhzhya regions, with frontlines stretching over 3,000 km, with 1000 km of active combat line and involving over 1,000,000 active Ukrainian combatants [11]. As a result, the daily life of the Ukrainian population is severely affected by continuous war-related stressors that can exacerbate their daily challenges [12]. The complex experience of war includes regular bombings far from the frontlines, disrupted access to essential services due to transportation and communication issues, increased poverty, unemployment, housing crisis, family separation, human rights violations, and other important stressors [12]. The conflict has resulted in significant loss of life. As of yet, it is estimated that the total number of casualties may approach 100,000 people [13,14]. Therefore, the unique challenges faced by the Ukrainian people amidst atrocities should be acknowledged when investigating the bereavement processes in the context of armed conflict.

Evidence from conflict-affected Ukrainian populations indicates that low mental well-being is widespread alongside elevated symptoms of depression, anxiety, and posttraumatic stress [15,16]. In addition to exposure to violence and loss, war-related socio-economic stressors, such as financial hardship, displacement, social isolation, and disruptions in family functioning, are likely to further undermine psychological well-being [17–19]. In line with the dual-continua model, examining well-being alongside psychopathology allows for a more differentiated understanding of mental health following bereavement in the context of armed conflict.

The current study aimed to further our understanding of mental health among Ukrainians bereaved during the armed conflict in Ukraine by adopting a person-centered perspective. Group-level averages and prevalence rates may obscure meaningful heterogeneity in mental health responses. Identifying person-centered patterns of co-occurring PG and PTS symptoms along with patterns of well-being, may therefore advance our understanding of bereavement-related mental health [20,21]. To illustrate, a sample of people who have experienced bereavement during the COVID-19 pandemic has been classified into four distinct categories using latent class analysis on dichotomous item-responses: low PGD/high well-being (32%), low PGD/moderate well-being (24%), moderate PGD/high well-being (23%), and high PGD/low well-being (21%) [9]. Identification of such subgroups highlights that symptoms of psychopathology and well-being can co-occur [22], underscoring the need for a person-centered approach to capture a complete picture of mental health outcomes following a loss in conflict-affected populations.

Furthermore, examining patterns of co-occurring indicators of psychopathology symptoms and well-being among bereaved Ukrainians may help to identify risk and protective factors associated with different mental health outcomes. Prior research has identified a range of factors associated with different bereavement outcomes. The intensity of PG symptoms is associated with risk factors, such as the loss of a partner or a child, the experience of loss due to violent circumstances [23], and a more recent time of loss [1]. Furthermore, older age is associated with lower psychopathology symptoms, but at the same time lower well-being [24]. Other factors such as exposure to other traumatic events [25], and forced displacement [26] have also been linked to the poorer mental health outcomes.

An additional factor that is key to mental health outcomes in conflict-affected populations is moral injury (MI) appraisals [21]. During armed conflicts, people may face moral transgressions, such as witnessing, perpetrating, or failing to prevent acts that violate their moral beliefs. This can have a lasting negative psychological, biological, spiritual, behavioral, and societal impact, depending on whether the moral transgression is attributed to one’s own actions or inaction (MI-self) or to the actions of others (MI-other) [25,27]. Prior literature has identified a strong association between more intense MI appraisals and more intense psychopathology symptoms, such as PTSD [21,25,28]. However, no study to date has examined the associations of MI appraisals and different patterns of co-occurring PG and PTS symptoms, and well-being.

### The current study

The ongoing Russian-Ukrainian war has had a significant impact on the mental health of the Ukrainian population [18,29–36]. In this context, war-related stressors may further exacerbate the emotional impact of bereavement [31]. The primary objective of this study was to examine distinct profiles of mental health based on International Classification of Diseases 11th Revision (ICD-11) PG symptoms, loss-related PTS symptom clusters of re-experiencing, avoidance, and hyperarousal, and subjective mental well-being among Ukrainian people who have experienced the loss of a close person during the Russian-Ukrainian war.

The prevalence of mental health difficulties in Ukraine poses a significant challenge to the nation’s already under-resourced mental health system [37–39]. Even prior to the full-scale invasion, funding for national mental health care facilities had been reduced, resulting in workforce losses and limited service capacity [40]. Specialist capacity has been limited, with approximately 11.6 psychiatrists and 1.15 psychologists per 100,000 people [41], compared to an average of 18.8 psychiatrists per 100,000 inhabitants across the European Union (EU-27) and the United Kingdom in 2021 [42]. Two years into the full-scale war in Ukraine, psychiatric hospitalizations have surpassed both pre-war levels and those recorded six months after the invasion. At the same time, the average number of psychiatrists, social workers, and junior nurses per facility has declined [43]. In addition, research among internally displaced and conflict-affected populations indicates that many individuals do not seek mental health support due to financial barriers, limited availability and quality of services, and stigma surrounding mental health problems [31]. Together, these system-level constraints and help-seeking barriers suggest that a substantial proportion of mental health needs remain unmet, underscoring the urgency of research that can inform targeted and efficient mental health responses.

In accordance with prior literature [1,9,23,25,44], our hypotheses were two-fold. First, we expected the presence of the following profiles: low symptoms and moderate/high well-being, moderate/high symptoms and moderate/high well-being, and moderate/high symptoms and low well-being. Second, we expected that people belonging to profiles with poorer mental health were more likely to have lost a partner or a child (vs. a more distantly related person), be more recently bereaved, be bereaved due to war-related violent circumstances (vs. other causes of death), have experienced more potentially traumatic events (PTE) in their lifetime, be forcibly displaced (vs. not displaced), and to report higher levels of MI appraisals.

## Methods

### Recruitment and procedure

Adult Ukrainians who had experienced the loss of a partner, family member, or friend were eligible to participate in this online survey study. The initial sample included 430 participants, but 140 respondents were excluded as they experienced the loss before February 24, 2022—the date of the full-scale invasion. Therefore, the final sample included 290 participants.

The survey was disseminated through various channels to reach a diverse sample of the Ukrainian population residing in Ukraine and The Netherlands. This included methods such as snowball sampling and the dissemination of a survey link through social media channels, including Facebook, Telegram, and WhatsApp groups aimed for Ukrainians. Additionally, flyers and posters were distributed in cafés, universities, and libraries in The Netherlands to reach people who had fled the war. Respondents were also recruited through contacts with Central Agencies for the Reception of Asylum Seekers (COAs), organizations engaged in refugee assistance, and people with extensive networks within the target population. Participation was limited to a single entry per individual and restricted to those accessing the survey through a direct Qualtrics link distributed via targeted channels; the survey was not publicly searchable or indexed by search engines. In addition, participants were asked whether they were interested in future research participation and whether they wished to receive study results via newsletter; those who responded affirmatively were invited to provide their email address. In total, 43% (n = 124) participants consented to future contact and 57% (n = 165) requested study results.

After completing the PG symptoms measure, participants received immediate feedback on their grief levels, categorized into four levels (green, yellow, orange, and red), each accompanied by a brief description of potential symptoms. Participants in the highest category were encouraged to seek professional support, such as consulting their general practitioner, and all participants were provided with a link to a Ukrainian website we developed that presents psychoeducation about grief (www.Вимірюваннягоря.com). This feedback was intended to provide informational support. The data collection period for this study spanned from January 2023 to July 2024. Informed consent was obtained digitally before participants accessed the survey. The Behavioral, Management and Social Sciences Ethics Committee at the University of Twente reviewed and approved the study (ID: 221111).

### Measures of mental health indicators

**PG symptoms.** The Traumatic Grief Inventory–Self-Report plus (TGI-SR+) is a 22-item questionnaire assessing PG symptoms severity [45]. Each item is rated on a scale from 1 (*never*) to 5 (*always*), an example item is “I found myself longing or yearning for the person who died”. Participants were asked to indicate how often they have experienced each reaction in the past month in response to the death of the loved one. Participants who had experienced the loss of multiple loved ones were instructed to keep in mind the loss that was the most distressing or frequently occupying their thoughts. Twelve TGI-SR+ items were selected that represent the 12 ICD-11 PG symptoms. A total score of ≥ 41 was used as a cut-off for clinically relevant ICD-11 PG symptom severity [45]. Throughout the manuscript, “clinically relevant” cut-offs refer to probable thresholds based on prior research and should not be interpreted as formal clinical diagnoses. The Ukrainian TGI-SR + was used which has shown adequate psychometric properties [46]. In the present study, the PG symptom items demonstrated high internal consistency (Cronbach’s α = .91).

**PTS symptoms.** The posttraumatic stress disorder checklist (PCL-5; [47,48]) short form was employed to assess PTS symptom severity across three symptom clusters based on ICD-11 criteria: re-experiencing, avoidance, and hyperarousal. Following prior research [49], this short form includes six items representing ICD-11 PTSD symptoms: item 2 (repeated, disturbing dreams), 3 (feeling or acting as if the experience were happening again), 6 (avoidance of internal reminders), 7 (avoidance of external reminders), 17 (being “superalert,” watchful or on guard) and 18 (feeling jumpy, easily startled). Participants rated how much each symptom bothered them in the previous month on 5-point Likert scales ranging from 0 (*not at all*) to 4 (*extremely*) in response to the loss of their loved one. A score of ≥ 3 indicated symptom presence, this cut-off score was consistent with the scoring approach used for PG symptoms [45], where the two highest response options on the scale indicated symptom presence. Thus, a total score of ≥ 18 was used as an approximated cut-off score for clinically relevant PTS symptom severity. In the present study, the internal consistency of the 6-item scale was adequate but modest (Cronbach’s α = .75) and in line with prior research [49].

**Well-being.** The 14-item Mental Health Continuum-Short Form (MHC-SF) was used to assess emotional, social, and psychological well-being [8]. Each item (e.g., “During the past month, how often did you feel happy?”) was rated from 0 (*never*) to 5 (*every day*). Mean scores below 2.6 indicate below-average well-being, as determined by the norm scores reported for the general population [50]. The MHC-SF has satisfactory psychometric properties [44], the internal consistency in the current sample was good (Cronbach’s α = .92 (total),  .88 (emotional),  .83 (social), and  .86 (psychological)).

### Measures of possible profile membership correlates

**Sociodemographic, loss, and war related characteristics.** Sociodemographic characteristics included age. Gender was not included as a predictor in the following analyses because the sample was predominantly female (95%), leaving insufficient variation for meaningful analysis. Loss-related characteristics comprised kinship to deceased (partner/child vs other), cause of death, and time since-loss (in months). Cause of death that was grouped into two categories: 1) death related to war (i.e., murder, manslaughter, and disappearance), 2) other (i.e., physical illness, sudden deaths not related to war). War-related characteristic included displacement status (being forcibly displaced either internally or externally vs not being displaced).

**Exposure to PTEs.** The Harvard Trauma Questionnaire (HTQ) Part I [51], is a 14-item checklist that assesses exposure to PTEs across the lifespan. Participants were asked to indicate whether they had experienced each event by responding “Yes” or “No” to the prompt: “Please indicate whether you have experienced any of the following events.” The total number of endorsed events was summed to create an index of cumulative trauma exposure.

**MI appraisals.** The Moral Injury Appraisals Scale (MIAS) is a 9-item questionnaire [25] that measures cognitive appraisals of MI. It consists of two subscales: MI-self and MI-other. MI-self refers to being troubled by moral transgressions of oneself (e.g., “I am troubled because I did things that were morally wrong”). MI-other refers to being troubled by moral violations of others (e.g., “I am troubled by morally wrong things done by other people”). Respondents were instructed to rate their agreement regarding each item on a scale from 1 (*strongly disagree*) to 4 (*strongly agree*). The MIAS demonstrated good internal consistency (Cronbach’s α = .83 (total);  .93 (MI-self);  .84 (MI-other)).

### Statistical analyses

LPA was conducted to identify latent subgroups. The identification of these profiles was based on response patterns to a set of continuous observed variables, i.e., indicators, namely self-reported total scores of PG symptoms, three PTS symptom clusters (i.e., re-experiencing, avoidance, and hyperarousal), and three types of well-being (i.e., emotional, social, and psychological). Indicators were z-standardized prior to analysis to place measures on a common scale and to focus on relative patterns across indicators. There were no missing data in terms of PG symptoms. PTS symptom ratings had 14% (n = 42) of missing data and well-being ratings had 31% (n = 89) missing data. The survey was divided into Part 1 and Part 2. Participants completed Part 1, which included sociodemographic and loss characteristics as well as the TGI-SR + , and received their personal grief outcome feedback. They were then explicitly asked if they wanted to continue with additional questions about the impact of loss during the war. Because not everyone chose to continue, this resulted in a considerable amount of missing data for variables collected in Part 2. Logistic regression results showed that participants who did vs. did not complete all items measuring PG symptoms, PTS, symptoms, and well-being did not differ in terms of sociodemographic and loss characteristics, with the exception of time since loss such that a longer time since the loss was associated with a higher likelihood of missing data (see S1 Table). Missing data on indicators were handled with full information maximum likelihood (FIML). Analyses were conducted in Mplus Version 8 [52]. The one-profile model was estimated first, followed by models with increasing numbers of profiles with the maximum of six. We used 100 random starts and 20 iterations.

The selection of the optimal solution was based on the following criteria: (a) lower values of the Akaike’s information criterion (AIC), Bayesian information criterion (BIC), and sample-size adjusted BIC (SA-BIC) indicate a better model fit [53]; (b) a *p*-value lower than  .05 for the bootstrapped likelihood ratio test (BLRt), the Lo-Mendell-Rubin adjusted likelihood ratio test (LMRt), and the Vuong-Lo-Mendell-Rubin adjusted likelihood ratio test (VLMRt), indicates that the model under consideration yields a significantly better fit than a model with one profile less; (c) higher entropy values (values closer to 1 indicate better profile separation) [53], d) adequate sample size within a latent profile (at least 5% of the total sample) [53], e) a meaningful interpretability of the profiles.

The correlates of profile-membership were examined using the three-step approach [54]. The three-step approach adjusts for the classification error when assigning people to profiles based on their highest probability estimate. We first conducted univariate multinomial logistic regressions with profile membership as the dependent variable and possible correlates considered in separate analyses. Then we performed a multivariate analysis including all correlates significantly associated with profile membership (*p* < .05) in the univariate models. The correlates included participant’s age, kinship, cause of death, time since loss, forced displacement, number of lifetime PTEs, and MI appraisals (i.e., MI-self, MI-other). Correlates of age, kinship, cause of death, and forced displacement did not have any missing data. Time since loss had 2% (n = 6) missing values, exposure to PTEs had 19% (n = 54) missing values, and MI appraisals had 26% (n = 76) missing values. To address missing data on correlates of profile membership, multiple imputation technique was applied, generating 100 imputed datasets [55,56]. The guidelines for reporting Latent Class Analysis (LCA) results (GRoLCA) were followed, which can also be applied to reporting LPA results [4].

### Open science practices

The analytic plan was pre-registered on the Open Science Framework: https://osf.io/jcv2y. The pre-registration took place after data collection, but before data analysis. The data of this project can be found on Data Archiving and Networked Services (DANS) repository under the link: https://doi.org/10.17026/SS/TWHH2D. Following preregistration but prior to analyzing the results, we refined our hypotheses. Originally, we planned to exploratively investigate associations between war-related factors, such as MI appraisals and forcible displacement (both internal and external), and the identified profiles. We then updated this to a directional hypothesis: we expected that people belonging to profiles characterized by poorer mental health would be more likely to have been forcibly displaced and to have higher levels of MI appraisals.

## Results

### Sample characteristics

In total, 290 participants reported to have lost a close person since February 22, 2024. The participants’ age ranged from 16 to 67 (M = 34.28, SD = 9.10) years. Most participants identified as female (95%) and had experienced the loss on average 5 months earlier, 38% experienced the loss at least six months ago. A total of 35% lost a partner or a child. The most common cause of loss was physical illness (43%), followed by war-related death (40%). Four out of five (81%) had experienced at least one traumatic event in their lifetime. The average PG symptom severity in this sample was 41.44 (*SD* = 8.39), 55% of participants scored above the cut-off for clinically relevant PG symptoms. The average loss-related PTS symptom severity in this sample was 8.67 (*SD* = 4.82), 4% of participants scored above the cut-off for clinically relevant PTS symptoms. The average item-scores of the three different subscales of well-being were between 1.94 and 2.58. Table 1 displays the sample characteristics.

**Table 1:** Participant characteristics and descriptives of PG symptoms, PTS symptoms, and well-being indicators (*N* = 290).

Characteristic	
Participant’s age, *M (SD)*, min-max	34.28 (9.10), 16-67
Gender, *N (%)*	
Female	269 (95)
Male	14 (5)
Other	0 (0)
Kinship, *N (%)*	
Partner	81 (28)
Child	19 (7)
Parent	73 (25)
Sibling	19 (7)
Grandparent	35 (12)
Friend	38 (13)
Other	25 (9)
Cause of death, *N (%)*	
Physical illness	125 (43)
Accident	22 (8)
Suicide	5 (2)
Homicide not related to war	2 (1)
Violent related to war (murder or manslaughter)	115 (40)
Disappearance	13 (4)
Other	8 (3)
Time since loss in months, *M (SD)*	5.28 (5.07)
Time since loss (categories), *N (%)*	
0-5 months	178 (62)
6-11 months	66 (23)
≥12 months	43 (15)
Forced displacement, *N (%)*	
No	174 (60)
Yes (within Ukraine)	30 (10)
Yes (abroad)	86 (30)
PTE lifetime exposure, *N (%)*	
No	44 (19)
Yes	192 (81)
Number of PTEs, *M (SD)*	2.59 (2.06)
MI appraisal, *M (SD)*	22.08 (5.98)
MI-self	10.35 (4.81)
MI-other	11.73 (3.01)
Mental health indicators, *M (SD)*, min-max	
PG symptoms	41.44 (8.39), 15-58
PTS symptoms	8.67 (4.82), 0-24
PTS: Re-experiencing	2.17 (1.80), 0-8
PTS: Avoidance	2.80 (2.29), 0-8
PTS: Hyperarousal	3.70 (2.17), 0-8
Well-being	2.26 (1.06), 0-5
Emotional well-being	1.94 (1.25), 0-5
Social well-being	2.06 (1.23), 0-5
Psychological well-being	2.58 (1.18), 0-5

MI = Moral injury, PTS = Posttraumatic stress, PTE = Potentially traumatic event, PG = Prolonged grief.

### Latent profiles of PG, PTS, and well-being

Table 2 shows the fit indices for the models including one through six profiles. The three-profile solution had the highest entropy value. The three-profile model had significant BLRt, VLMRt and LMRt *p* values (*p* < .05), indicating that these models had a significant improvement in fit compared to the two-profile model. In contrast, the four-profile solution had non-significant VLMRt and LMRt *p* values. Based on the fit statistics and parsimony of the profile solutions, the three-profile model was retained. Fig 1 illustrates the three-profile model (see S2 Fig. for alternative profile solution figures).

**Table 2:** Fit indices of one to six latent profiles models (*N* = 290).

Model	Log Likelihood	BIC	AIC	SA-BIC	Entropy R²	BLRt *p*-value	VLMRt *p*-value	LMRt *p*-value	Sample size per profile
**One profile**	−2319.30	4717.97	4666.59	4673.57	–	–	–	–	–
**Two profiles**	−2165.34	4455.41	4374.67	4385.65	.671	< .001	.011	.012	168/122
**Three profiles**	−2112.11	4394.31	4284.22	4299.18	.762	< .001	.005	.005	165/87/38
**Four profiles**	−2071.70	4358.85	4219.39	4238.34	.689	< .001	.293	.300	89/85/74/41
**Five profiles**	−2047.11	4355.04	4186.22	4209.16	.680	< .001	.090	.093	86/71/55/45/33
**Six profiles**	−2033.75	4373.67	4175.49	4202.42	.667	.050	.452	.458	77/50/49/45/36/33

AIC = Akaike Information Criterion, BIC = Bayesian Information Criterion, BLRt = Bootstrap Likelihood Ratio test, LMRt = Lo-Mendell-Rubin adjusted likelihood ratio test, SA-BIC = Sample Size Adjusted Bayesian Information Criterion, VLMRt = Vuong-Lo-Mendell-Rubin adjusted likelihood test.

**Fig 1:**
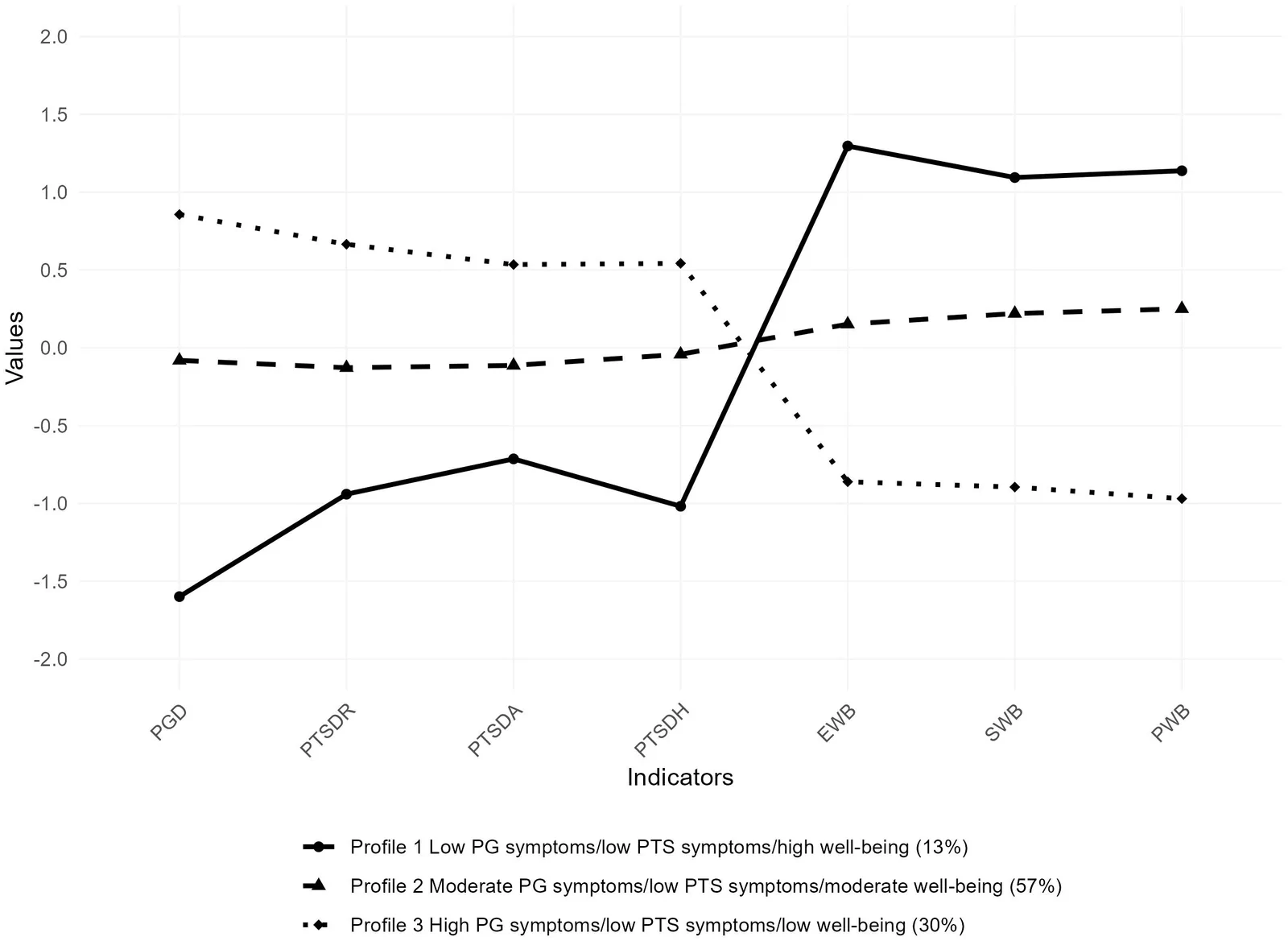
Three-profile model for PG symptoms, PTS symptoms, and well-being profiles (*N* = 290). EWB = Emotional well-being, PGD = Prolonged grief disorder, PTSDA = Posttraumatic stress disorder Avoidance cluster, PTSDH = Posttraumatic stress disorder Hyperarousal cluster, PTSDR = Posttraumatic stress disorder Re-experiencing cluster, PWB = Psychological well-being, SWB = Social well-being.

Table 3 presents unstandardized PG, PTS, and well-being means of each profile. The first and smallest profile included 38 participants (13%) showing PG and PTS symptoms that were below clinically relevant levels and above average well-being of all three types. This profile was labeled as ‘low PG symptoms/low PTS symptoms/high well-being’. The second and largest profile consisted of 165 participants (57%) with moderate, clinically relevant levels of PG, but low PTS symptoms, and moderate levels of well-being. Almost half of the participants (42%) that belonged to this profile scored above the clinical cut-off for PGD, but only 1% scored above the clinical cut-off for PTSD. This profile was called ‘moderate PG symptoms/low PTS symptoms/moderate well-being’. The third profile included 87 participants (30%) that evidenced high, above clinical cut-off PG symptoms, low PTS symptoms and low well-being scores. In this profile, 93% of people scored above the clinical cut-off of PGD, and 11% above the clinical cut-off of PTSD, more than 90% of participants scored below the well-being threshold. This profile was labeled ‘high PG symptoms/low PTS symptoms/low well-being’.

**Table 3:** Descriptives associated with each profile in the three-profile solution (*N* = 290).

	Possible range	Profile 1 Low PG symptoms/low PTS symptoms/high well-being *N* = 35 (12%)	Profile 2 Moderate PG symptoms/low PTS symptoms/moderate well-being *N* = 171 (59%)	Profile 3 High PG symptoms/low PTS symptoms/low well-being *N* = 84 (29%)
PG symptoms, *M (SD)*	12-60	27.06 (4.75) (0% met PGD criteria)	40.58 (5.51) (42% met PGD criteria)	49.19 (4.93) (93% met PGD criteria)
PTS symptoms, *M (SD)*	0-24	2.91 (2.48) (0% met PTSD criteria)	7.96 (3.83) (1% met PTSD criteria)	12.38 (4.20) (10.5% met PTSD criteria)
PTS: Re-experiencing	0-8	0.41 (0.67)	1.93 (1.53)	3.34 (1.83)
PTS: Avoidance	0-8	1.09 (1.28)	2.49 (2.13)	4.08 (2.27)
PTS: Hyperarousal	0-8	1.41 (1.54)	3.54 (1.94)	4.96 (1.90)
Well-being, *M (SD)*	0-5	3.67 (0.54)	2.55 (0.69)	1.11 (0.56)
Emotional well-being	0-5	3.63 (0.75) (92% ≥ 2.6)	2.15 (0.97) (37% ≥ 2.6)	0.83 (0.74) (95% < 2.6)
Social well-being	0-5	3.38 (0.73) (85% ≥ 2.6)	2.36 (1.09) (43% ≥ 2.6)	0.95 (0.66) (98% < 2.6)
Psychological well-being	0-5	3.94 (0.64) (96% ≥ 2.6)	2.90 (0.85) (65% ≥ 2.6)	1.39 (0.81) (89% < 2.6)

PG = Prolonged grief; PGD = Prolonged grief disorder; PTS = Posttraumatic stress; PTSD = Posttraumatic stress disorder.

### Correlates of profile membership

Multinomial regression analyses were conducted to examine the correlates of profile membership. Table 4 presents the observed means and frequencies for all correlates across profiles. Table 5 presents the multinomial regression results. First, univariate analyses indicated that compared to ‘low PG symptoms/low PTS symptoms/high well-being’ and ‘moderate PG symptoms/low PTS symptoms/high well-being’ profiles, people in ‘high PG symptoms/low PTS symptom/low well-being’ profiles were more likely to have experienced the loss of a child or partner (compared to other types of loss), to have lost a loved one due to war-related violence, and to have been forcibly displaced. Second, in the multivariate model, only kinship and forced displacement remained significant correlates. No significant differences emerged between the ‘low PG symptoms/ low PTS symptoms/high well-being’ and the ‘moderate PG symptoms/low PTS symptoms/moderate well-being’ profiles based on any of the examined correlates.

**Table 4:** Sociodemographic, Loss, and War-related Characteristics of Each Profile (*N* = 290).

	Profile 1 Low PG symptoms/low PTS symptoms/high well-being *N* = 35 (12%)	Profile 2 Moderate PG symptoms/low PTS symptoms/moderate well-being *N* = 171 (59%)	Profile 3 High PG symptoms/low PTS symptoms/low well-being *N* = 84 (29%)
Participant’s age (in years), *M (SD)*	36.91 (9.95)	33.84 (8.60)	34.10 (9.64)
Kinship, *N (%)*			
Child or partner	3 (9%)	45 (26%)	52 (62%)
Other	32 (91%)	126 (74%)	32 (38%)
Cause of death, *N (%)*			
War related	9 (26%)	70 (41%)	49 (58%)
Other	26 (74%)	101 (59%)	35 (42%)
Time since loss (in months), *M (SD)*	5.18 (5.40)	4.90 (4.53)	6.11 (5.88)
Forced displacement, *N (%)*			
Yes	10 (27%)	61 (36%)	45 (54%)
No	25 (71%)	110 (64%)	39 (46%)
Number of PTEs, *M (SD)*	2.33 (2.41)	2.52 (1.99)	2.84 (2.02)
MI appraisal^b^, *M (SD)*			
MI-self	10.00 (4.91)	9.82 (4.64)	11.45 (4.97)
MI-other	10.86 (2.72)	11.72 (3.01)	12.11 (3.10)

MI = Moral injury, PG = Prolonged grief, PTE = Potentially traumatic event, PTS = Posttraumatic stress. ^**a**^N = 236. ^**b**^N = 214.

**Table 5:** Multinomial Logistic Regression Estimates of Correlates of Profile Membership (*N* = 290).

	Low PG symptoms/low PTS symptoms/high well-beingvs.Moderate PG symptoms/low PTS symptoms/moderate well-being			Low PG symptoms/low PTS symptoms/high well-beingvs.High PG symptoms/low PTS symptoms/low well-being			Moderate PG symptoms/low PTS symptoms/moderate well-beingvs. High PG symptoms/low PTS symptoms/low well-being		
*Univariate analyses*	b	SE	*p*	b	SE	*p*	b	SE	*p*
Participant’s age	−0.039	0.022	.074	−0.033	0.023	.154	0.006	0.020	.765
Kinship(0 = Other; 1 = Partner or child)	1.382	0.833	.097	3.266	0.822	**.000**	1.884	0.386	**.000**
Cause of death(0 = Other; 1 = Related to war)	0.720	0.495	.146	1.568	0.506	**.002**	0.848	0.348	**.015**
Time since loss (in months)	−0.018	0.045	.696	0.039	0.046	.387	0.057	0.032	.072
Forced displacement(0 = No, 1 = Yes)	0.289	0.477	.545	1.190	0.483	**.014**	0.901	0.348	**.010**
Number of PTEs	0.091	0.176	.607	0.166	0.166	.317	0.076	0.085	.371
MI appraisal									
MI-self	−0.021	0.055	.706	0.059	0.055	.282	0.080	0.041	.052
MI-other	0.097	0.072	.182	0.157	0.083	.060	0.060	0.076	.432
*Multivariate analyses*									
Kinship(0 = Other; 1 = Partner or child)	1.367	0.889	.124	3.616	0.981	**.000**	2.249	0.505	**.000**
Cause of death(0 = Other; 1 = Related to war)	0.535	0.507	.291	0.983	0.582	.092	0.447	0.411	.276
Forced displacement	0.493	0.500	.324	2.074	0.643	**.001**	1.580	0.488	**.001**

MI = Moral injury, PG = Prolonged grief, PTE = Potentially traumatic event, PTS = Posttraumatic stress, SE = standard error. Bold values represent significant findings

## Discussion

To the best of our knowledge, this is the first study to examine mental health profiles in bereaved people in the context of international armed conflict. Using LPA, we identified three distinct mental health profiles in a sample of Ukrainians bereaved after the full-scale Russian invasion in 2022. One in 10 people experienced low PG and PTS symptoms and high well-being. Six in 10 people experienced moderate PG, low PTS symptoms, and moderate well-being. Three in 10 people experienced high PG, low PTS symptoms, and low well-being.

The dual continua model of mental health suggests that higher PG and PTS symptoms can coexist with moderate to high well-being, and vice versa, that low well-being can be associated with low to moderate PG and PTS symptoms [9,44]. Contrary to our expectations and previous findings in people bereaved during the COVID-19 pandemic [9], the current results do not appear to support the assumptions of the dual continua model. The current study showed that the largest group of people reported moderate levels of well-being while experiencing moderate levels of PG symptoms. Two other profiles emerged in addition to this group: One characterized by high PG, low PTS symptoms and low well-being and another with low PG and low PTS symptoms and high well-being. These profiles do not provide evidence for a profile combining high symptomatology with high well-being, as would be expected under the dual continua model. However, given the cross-sectional design, this finding should be interpreted with caution, as relationships between symptomatology and well-being may vary over time.

A scoping review by Iasiello et al. [44] suggests that at higher levels of psychopathology, such as depression, people may struggle to accurately evaluate their well-being due to the high level of distress. For instance, preconceived beliefs about how distress relates to well-being, such as thinking that high distress means one cannot be truly well, may distort people’s self-assessments of their positive mental health. More than half of participants scored above the cut-off for clinically relevant PG symptoms which can potentially account for the limited support for the dual continua model in the context of PG symptoms. However, findings related to PTS symptoms and well-being appear more in line with the dual continua model: While well-being varied across profiles, PTS symptoms remained consistently low. This suggests that PTS and well-being may operate on two separate continua, as the model proposes, whereas PG symptoms and well-being appear more tightly linked in our sample.

Moreover, the distribution of the three profiles differed from that in earlier studies, which typically identified a low-symptom and high well-being group as the largest [9,44], a pattern also supported by grief trajectory studies [1,57,58]. In contrast, low-symptom and high well-being group was the smallest in our sample, suggesting that Ukrainians that were bereaved during the ongoing armed conflict may face unique mental health challenges. A variety of war-related stressors can hinder people’s ability to sustain well-being under such external pressures [12,31,36]. These findings emphasize the importance of context-sensitive approaches in understanding and supporting bereaved populations affected by conflict.

This sample of bereaved Ukrainians who are confronted with an ongoing armed conflict showed elevated levels of PG but not of bereavement-related PTS symptoms. This pattern supports the notion that PGD and PTSD are related yet distinct conditions in bereaved people [6]. While previous research has reported high PTSD prevalence among Ukrainian civilians following the invasion (e.g., 27% and 74%) [28], these studies typically assessed various types of traumatic experiences. In contrast, our study focused specifically on bereavement-related PTS symptoms, which may explain the discrepancy. Notably, many participants experienced significant psychological distress that would have gone undetected if PG symptoms had not been assessed. Our findings confirm the need to distinguish between different forms of post-loss psychopathology.

We examined the associations between profile membership and various correlates, including age, kinship, cause of death, time since loss, forced displacement, number of PTEs, and MI appraisals. Experiencing the loss of a partner or child and being forcibly displaced were associated with a higher likelihood of belonging to the profile characterized by high PG, low PTS symptoms, and low well-being. The loss of a close relationship, i.e., a partner or child, can elicit intense separation distress, which lies at the core of PG symptomatology, and is a consistently identified risk factor for elevated PG symptoms [23,57]. Additionally, our findings align with previous findings that showed forced displacement can negatively impact mental health, potentially due to compounding stressors, such as ongoing uncertainty, separation from loved ones, and diminished control over one’s life [21,29,59,60].

Interestingly, MI appraisal ratings did not differ significantly between the identified profiles. The growing body of evidence suggests that stronger MI appraisals are associated with greater PTS symptom severity [21]. However, in the current study PTS symptoms remained low in all three profiles, which may explain our null-finding. Moreover, a previous study of Ukrainian civilians reported relatively weak associations between MI severity and PTSD symptoms, suggesting that the relationship may vary by context and population [28].

The findings of this study highlight an important clinical implication. Clinically relevant PG symptoms can go unnoticed without proper assessment. However, access to mental healthcare remains limited for many Ukrainians due to displacement, structural barriers, and ongoing conflict. In this context, scalable, low-threshold interventions that reduce PG symptoms and promote well-being are important. Examples include group-based acceptance and commitment therapy programs such as Self-Help Plus (SH+), “Doing What Matters in Times of Stress,” and digital tools like the chatbot “Friend,” which are being implemented among Ukrainian populations [51,55,56]. These efforts, including initiatives like Reach out, Implement, Scale-up, and Evaluate (U-RISE), represent promising avenues to extend support to underserved groups [57].

### Strengths and limitations

This is one of the first studies to examine the mental health following bereavement in the context of armed conflict. Data collection for this study took place from January 2023 to July 2024, capturing an extended timeframe of the ongoing armed conflict. This extended timeframe may reflect a period of psychological accommodation for some people, while simultaneously increasing the risk of chronic distress due to sustained exposure to trauma, displacement, and societal exhaustion. The current study also shifts the focus from mental illness to mental health, by using a person-centered approach that includes indicators of both psychopathology and well-being.

Nevertheless, the findings should be interpreted in light of a few limitations. First, our findings pertain only to elevated early PG symptoms rather than a formal diagnosis of PGD, as most participants had lost a loved one within the past 5 months – the minimum time criterion for a PGD diagnosis according to ICD-11. However, prior research indicates that early PG symptoms are strong predictors of later clinically relevant PGD [61], supporting the importance of early screening and intervention [62]. Second, this study did not control for the specific war-related stressors, such as disrupted access to essential resources, unemployment, housing crisis, family separation, experienced by the bereaved. Third, the mental health profiles were assessed cross-sectionally, for this reason, the stability of these profiles remains unknown. Future research could address this by introducing continuous follow-up measures and using latent transition or trajectory analyses [1,63]. Fourth, missing data on profile indicators (PG symptoms, PTS symptoms, and well-being) were handled using FIML estimation, whereas missing covariate data were addressed using multiple imputation. Although participants with complete and incomplete data did not differ on most sociodemographic or loss-related characteristics (except time since loss), self-selection bias remains a potential limitation. Fifth, the sample was predominantly female (95%), reflecting a common gender imbalance in bereavement research and limiting generalizability to male bereaved individuals. This imbalance may partly reflect the war context, as many men in Ukraine are drafted or volunteering for military service, reducing their availability for study participation. Finally, although data quality checks were applied, additional procedures to verify eligibility and prevent fraudulent responses (e.g., attention and consistency checks or geolocation verification) were not implemented and should be considered in future research using online recruitment, particularly in light of the rapidly evolving digital environment.

## Conclusion

In conclusion, three distinct mental health subgroups emerged among Ukrainians who lost a loved one during the full-scale Russian invasion. Most bereaved people in this study exhibited moderate levels of symptoms and moderate levels of well-being, while only a minority reported low symptoms and high well-being. This study highlights a unique sample where PG and well-being seem to lie on opposite ends of the same continuum rather than distinct dimensions, providing limited support for the dual-continua model. Participants primarily experienced PG symptoms, while loss-related PTS symptoms were less prominent. Furthermore, losing a partner or child and being forcibly displaced were associated with greater psychological distress and lower well-being. Identifying different mental health profiles helped clarify which groups need more support, emphasizing the importance of making scalable interventions that target both PG symptoms and well-being more widely accessible.

## Supporting information

S1 Table Multivariate binary logistic comparing participants with complete data on all indicators and participants who had missing data based on sociodemographic and loss characteristics. (PDF)

S1 Fig Figures for alternative profile solutions. One, two, four, five, and six profile models.
(PDF)
