# Supplementary material for: Profiles of mental health indicators among Ukrainian people recently bereaved in the context of the full-scale Russian invasion
Source: PLoS One. 2026 Nov 3;21(11):e0358123. doi: 10.1371/journal.pone.0358123 (PMC13730338; doi:10.1371/journal.pone.0358123)
Supplement: Supplementary file 1 [file pone.0358123.s001.pdf]

Supplementary material

Profiles of mental health indicators among Ukrainian people recently bereaved in the context of full-scale Russian invasion

Justina Pociūnaitė-Ott, Paul A. Boelen, Iryna Frankova, Iryna Norkina, Maria Luisa F. Rispa Hoyos, Lonneke I.M. Lenferink

S1 Table

Multivariate binary logistic comparing participants with complete data on all indicators and participants who had missing data based on sociodemographic and loss characteristics

**Variable**	**B (SE)**	***p* - value**	**OR**	**95% CI**
Age	-0.02 (0.02)	.269	0.97	[0.93, 1.02]
Kinship				
(0 = Other; 1 = Partner or child)	0.36 (0.42)	.383	1.44	[0.63, 3.27]
Cause of death				
(0 = Other; 1 = Violent due to war)	-0.77 (0.42)	.067	0.47	[0.2, 1.03]
Time since loss (in months)	0.11 (0.04)	.002	1.12	[1.04, 1.21]
Forced displacement				
(0 = No; 1 = Yes)	0.72 (0.39)	.067	2.05	[0.95, 4.46]
PTE count	-0.13 (0.11)	.237	0.88	[0.69, 1.08]

PTE = Potentially traumatic event.
