# Supplementary material for: Profiles of mental health indicators among Ukrainian people recently bereaved in the context of the full-scale Russian invasion
Source: PLoS One. 2026 Nov 3;21(11):e0358123. doi: 10.1371/journal.pone.0358123 (PMC13730338; doi:10.1371/journal.pone.0358123)

Supplementary material

Profiles of mental health indicators among Ukrainian people recently bereaved in the context of full-scale Russian invasion

Justina Pociūnaitė-Ott, Paul A. Boelen, Iryna Frankova, Iryna Norkina, Maria Luisa F. Rispa Hoyos, Lonneke I.M. Lenferink

S2 Fig.

EWB = Emotional well-being, PGD = Prolonged grief disorder, PTSDA = Posttraumatic stress disorder Avoidance cluster, PTSDH = Posttraumatic stress disorder Hyperarousal cluster, PTSDR = Posttraumatic stress disorder Re-experiencing cluster, PWB = Psychological well-being, SWB = Social well-being.

Figure S1

One-profile model for PG symptoms, PTS symptoms, and well-being profiles (N = 290)

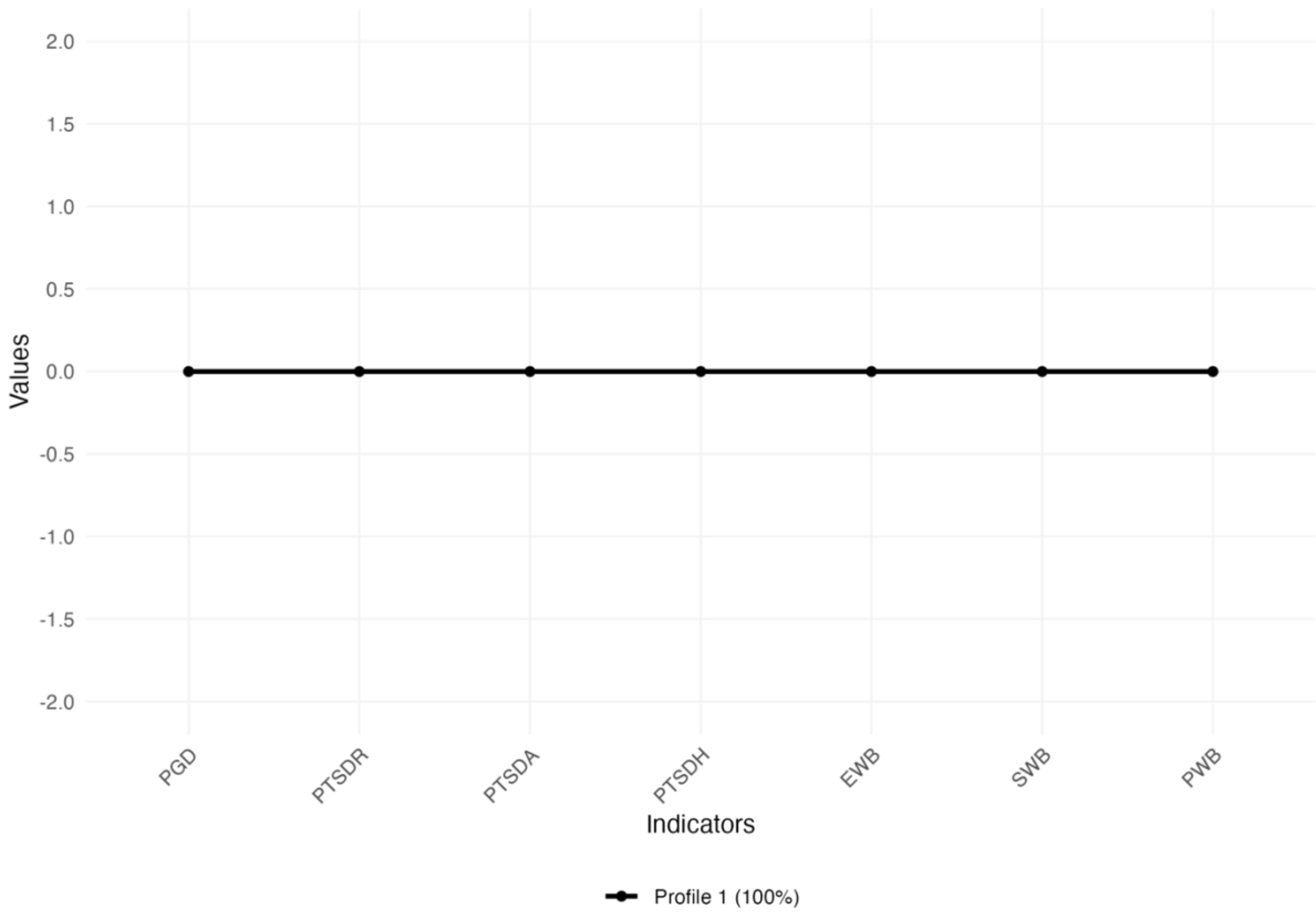

Figure S2

Two-profile model for PG symptoms, PTS symptoms, and well-being profiles (N = 290)

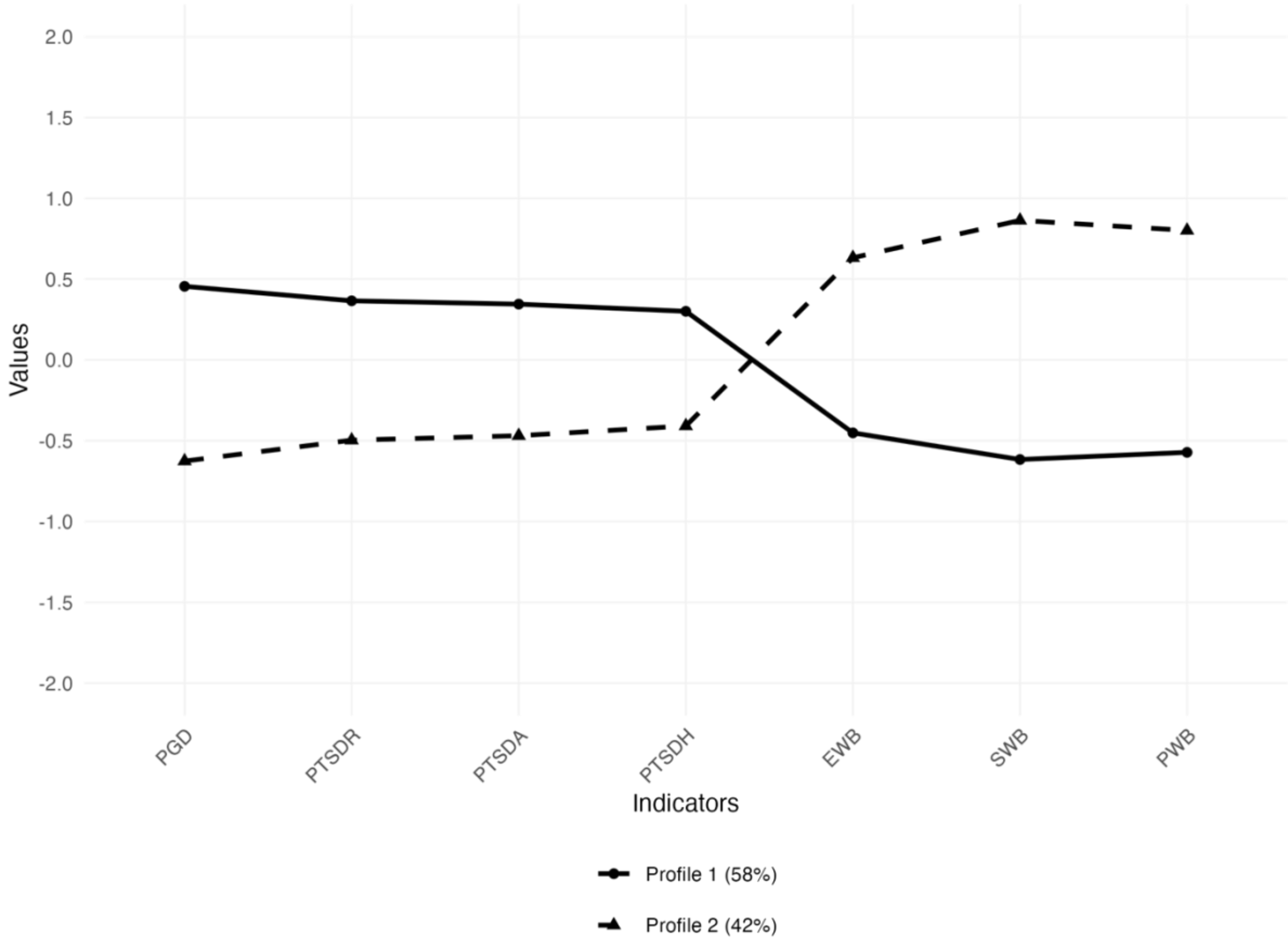

Figure S3

Four-profile model for PG symptoms, PTS symptoms, and well-being profiles (N = 290)

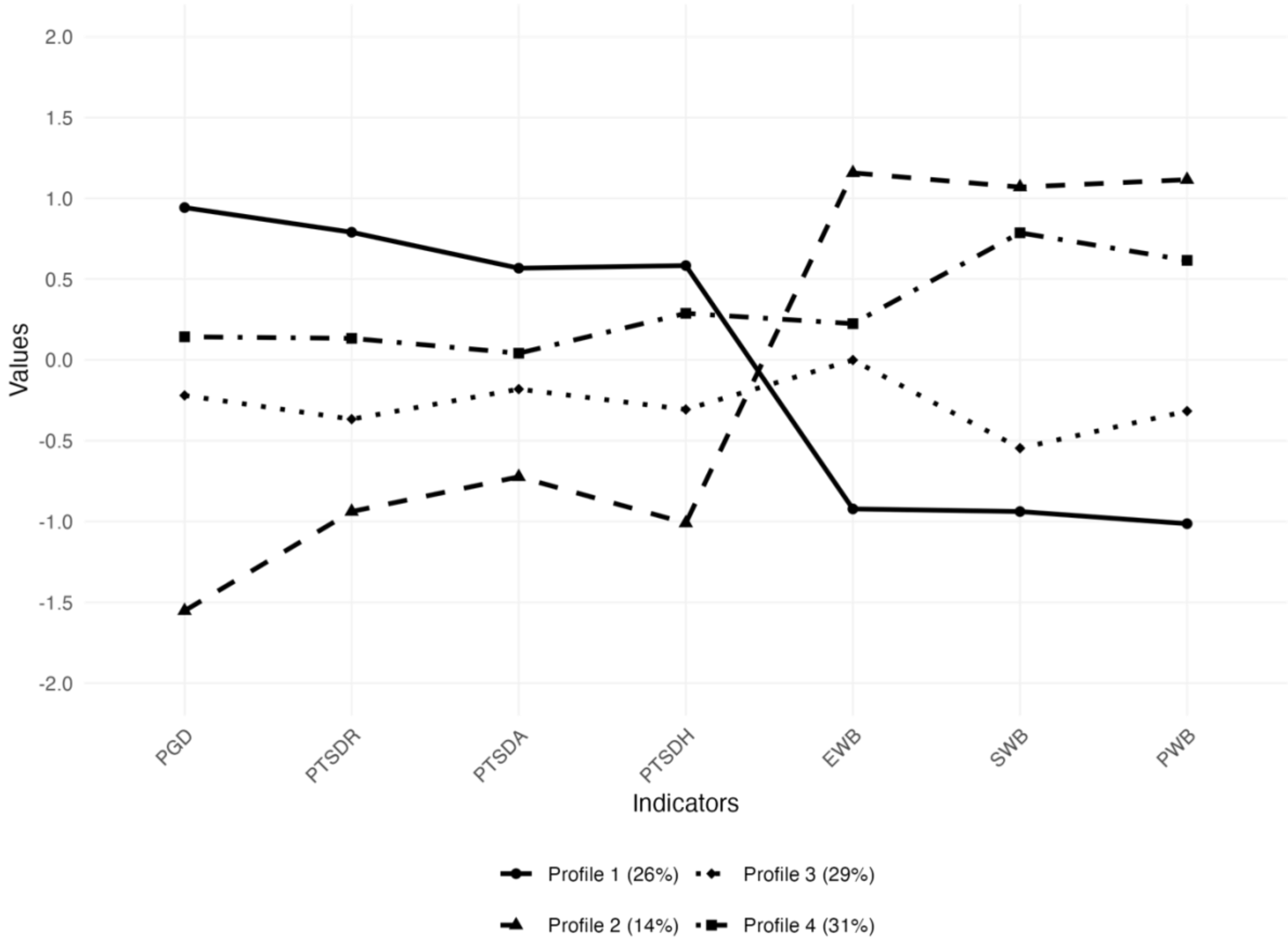

Figure S4

Five-profile model for PG symptoms, PTS symptoms, and well-being profiles (N = 290)

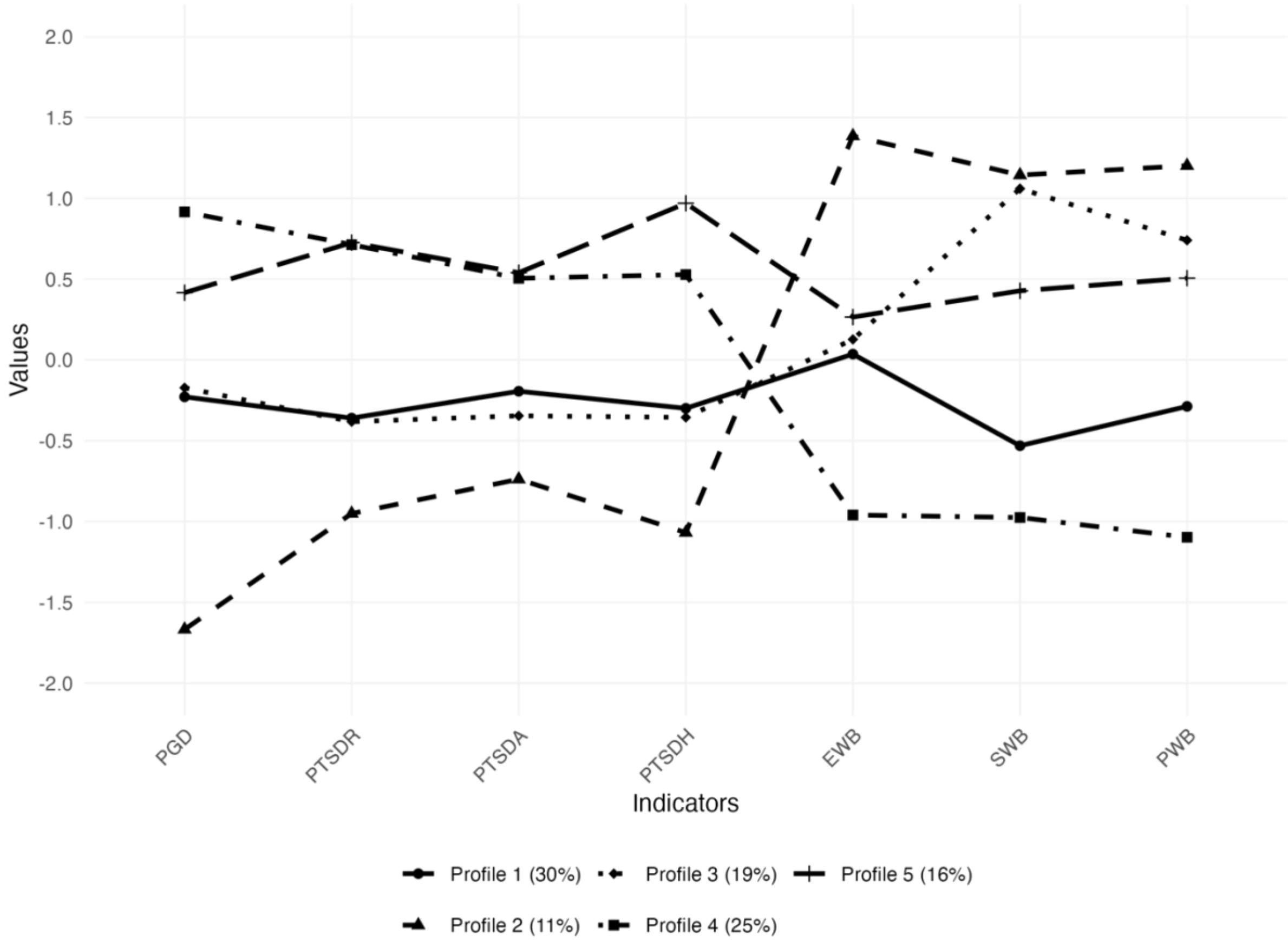

Figure S5

Six-profile model for PG symptoms, PTS symptoms, and well-being profiles (N = 290)

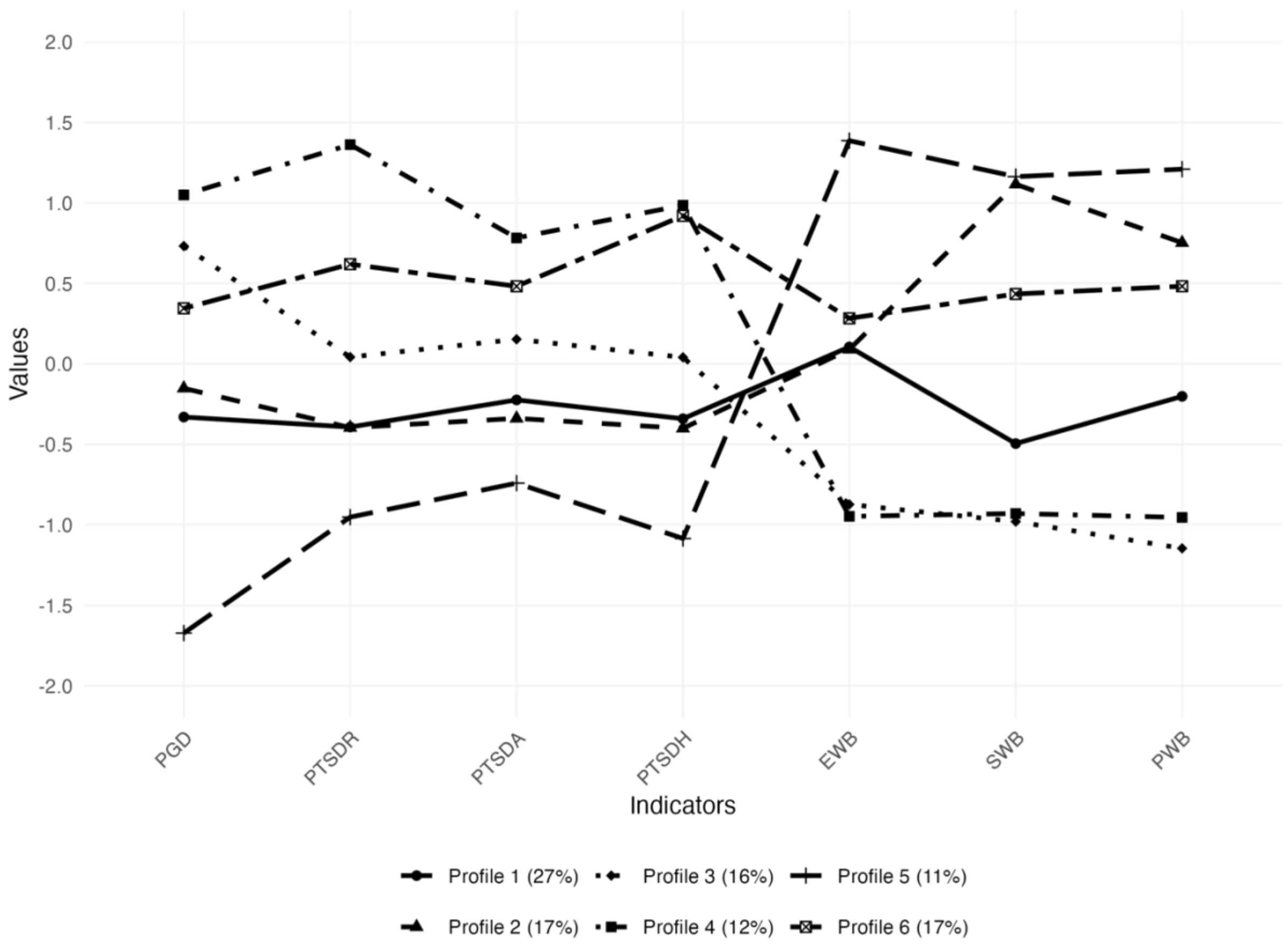

Supplement: Supplementary file 2 [file pone.0358123.s002.pdf]
