# Supplementary material for: Profiles of mental health indicators among Ukrainian people recently bereaved in the context of the full-scale Russian invasion
Source: PLoS One. 2026 Nov 3;21(11):e0358123. doi: 10.1371/journal.pone.0358123 (PMC13730338; doi:10.1371/journal.pone.0358123)
Supplement: Supplementary file 3 [file pone.0358123.s003.docx]

Response to Reviewers

Reviewer #1:

This is an important and timely study addressing the mental health of bereaved Ukrainians during the conflict. The focus on both prolonged grief and well-being is valuable, and the person-centered approach is a strength. The manuscript is generally well-written and clear.

**Response:**
We thank the reviewer for their positive evaluation of the study and for highlighting the relevance of the topic, the inclusion of both prolonged grief and well-being, and the use of a person-centered approach.

**Comment:**
However, there are some suggestions that could strengthen this paper:
1. The introduction suggests that well-being in bereavement has been largely neglected. However, several recent studies have examined well-being alongside grief (e.g., Alpysbekova A., Cisco, M. M., Beyhan Ertanir, Vo, D. H., Scaramutti, C., Nehme, L., Montero-Zamora, P., Bautista, T., & Schwartz, S. J. (2024). Cultural–economic stress and mental health among Ukrainian immigrants residing in the U.S. post-Russian invasion. American Journal of Orthopsychiatry. <https://doi.org/10.1037/ort0000796>; Alpysbekova A., Cisco, M. M., Vo, D. H., Beyhan Ertanir, Sumeyra Sahbaz, Montero-Zamora, P., Bautista, T., Scaramutti, C., Bartoszak, M., Nehme, L., Duque, M., & Schwartz, S. J. (2024). The effect of the Russian invasion on mental, social, and behavioral health among Ukrainians living in the United States. Psychological Trauma Theory Research Practice and Policy. <https://doi.org/10.1037/tra0001770>; Alpysbekova A., Scaramutti-Gladfelter, C., Cisco, M. M., Vo, D. H., Duque, M., Watkins, L. G., Cobb, C. L., Lee, S. W., Montero-Zamora, P., Schwartz, S. J., & Inna Pokul. (2025). Anticipatory grief and perseverative worry: Psychological impacts on Ukrainian migrants from the 2022 Russian invasion. Psychological Trauma Theory Research Practice and Policy. <https://doi.org/10.1037/tra0002024>; Duque, M., Vo, D. H., Montero-Zamora, P., Cobb, C. L., Sahbaz, S., Ertanir, B., Bautista, T., Watkins, L. G., Acaf, Y., Macias, M. A., Alpysbekova, A., Garcia, M. F., Rodriguez, J., Bates, M. M., Calderon, I. A., Maldonado-Molina, M. M., Bartholomew, J. B., Pinedo, M., Lee, T. K., Salas-Wright, C. P., & Schwartz, S. J. (2025). Longitudinal determinants of thriving in Puerto Rican climate crisis migrants on the U.S. mainland: A structural equation model. Social Science & Medicine, 366, Article 117615. <https://doi.org/10.1016/j.socscimed.2024.117615>; ).
This gap statement should be softened or more accurately specified.

**Response:**
We thank the reviewer for this suggestion. In response, we revised the manuscript to soften and more precisely specify the gap regarding well-being in bereavement.

Abstract:

*Bereavement research has frequently focused on psychopathology, while comparatively less attention has been given to well-being as a component of mental health.*

Page 3:
*Relatively little attention has been given to mental health after bereavement, particularly in terms of both psychopathology and well-being as coexisting dimensions, and when the loss occurs during an international armed conflict.*

We also incorporated and cited the suggested literature at relevant points in the manuscript.

Page 4:
*In addition to exposure to violence and loss, war-related socio-economic stressors, such as financial hardship, displacement, social isolation, and disruptions in family functioning, are likely to further undermine psychological well-being (17–19).*

Page 24:
*Additionally, our findings align with previous findings showed that forced displacement can negatively impact mental health, potentially due to compounding stressors, such as ongoing uncertainty, separation from loved ones, and diminished control over one’s life (21,30,60,61).*

**Comment:**
2. The technical description of LPA feels premature here and distracts from establishing the theoretical and empirical context. It would be more appropriate in the Methods/Data Analysis section.

**Response:**
We substantially revised the Introduction to reduce technical and statistical details related to LPA. Methodological explanations have been removed from the introduction, and it now focuses on the broader conceptual relevance of person-centered approaches. All technical and procedural details of the LPA are presented in the Methods/Data Analysis section.

Pages 4-5:
*The current study aimed to further our understanding of mental health among Ukrainians bereaved during the armed conflict in Ukraine by adopting a person-centered perspective.*

Page 5:
*Furthermore, examining patterns of co-occurring indicators of psychopathology symptoms and well-being among bereaved Ukrainians may help to identify risk and protective factors associated with different mental health outcomes.*

**Comment:**
3. Rather than explaining how LPA works, the introduction should explain why a person-centered approach is needed (e.g., heterogeneity in bereavement responses, coexistence of psychopathology and well-being). This argument could be supported with appropriate citations.

**Response:**
In line with this suggestion, we revised the Introduction to more clearly articulate why a person-centered approach is important in the context of bereavement research. The revised text emphasizes heterogeneity in bereavement responses and the coexistence of psychopathology and well-being as key arguments for this approach, and we added relevant citations to support this argument. Only a brief, non-technical reference to group-based analyses was retained to motivate the study aims.

Page 5:
*Group-level averages and prevalence rates may obscure meaningful heterogeneity in mental health responses. Identifying person-centered patterns of co-occurring PG and PTS symptoms, along with patterns of well-being, may therefore advance our understanding of bereavement-related mental health (20,21).*

**Comment:**
4. Before introducing the LPA, it would help to explain why well-being is essential in the current research: prevalence rates, existing findings in conflict settings, and what is currently known about well-being among bereaved Ukrainians. Right now, the justification feels brief compared to the detail given to psychopathology.

**Response:**
We expanded the Introduction to more clearly justify the inclusion of well-being alongside psychopathology prior to introducing the person-centered analyses.

Page 4:
*Evidence from conflict-affected Ukrainian populations indicates that low mental well-being is widespread alongside elevated symptoms of depression, anxiety, and posttraumatic stress (15,16). In addition to exposure to violence and loss, war-related socio-economic stressors, such as financial hardship, displacement, social isolation, and disruptions in family functioning, are likely to further undermine psychological well-being (17–19). In line with the dual-continua model, examining well-being alongside psychopathology allows for a more differentiated understanding of mental health following bereavement in the context of armed conflict.*

**Comment:**
5. The introduction mentions that mental health difficulties pose a challenge to the Ukrainian system, but presenting concrete statistics (prevalence, service gaps, burden of need) would strengthen the urgency and relevance of the study

**Response:**
We revised the Introduction to strengthen the contextualization of mental health challenges in Ukraine by incorporating available evidence on mental health needs and service strain in conflict-affected settings.

Page 6:
*Even prior to the full-scale invasion, funding for national mental health care facilities had been reduced, resulting in workforce losses and limited service capacity (41). Specialist capacity remains constrained, with approximately 11.6 psychiatrists and 1.15 psychologists per 100,000 people (42), compared to an average of 18.8 psychiatrists per 100,000 inhabitants across the European Union (EU-27) and the United Kingdom in 2021 (43). Two years into the full-scale war in Ukraine, psychiatric hospitalizations have surpassed both pre-war levels and those recorded six months after the invasion. At the same time, the average number of psychiatrists, social workers, and junior nurses per facility has declined (44). In addition, research among internally displaced and conflict-affected populations indicates that many individuals do not seek mental health support due to financial barriers, limited availability and quality of services, and stigma surrounding mental health problems (32). Together, these system-level constraints and help-seeking barriers suggest that a substantial proportion of mental health needs remain unmet, underscoring the urgency of research that can inform targeted and efficient mental health responses.*

**Comment:**
6. In the present study Aims and hypotheses should be stated clearly, succinctly, and separately. I recommend clearly separating background literature from the study’s aims and hypotheses for improved clarity and flow.

**Response:**
We revised the manuscript to separate the background literature from the study’s aims and hypotheses. The aims and hypotheses are now presented in a distinct section and stated more succinctly to improve clarity and flow.

Page 7:
*In accordance with prior literature (1,9,24,27,44), our hypotheses were two-fold. First, we expected the presence of the following profiles: low symptoms and moderate/high well-being, moderate/high symptoms and moderate/high well-being, and moderate/high symptoms and low well-being. Second, we expected that people belonging to profiles with poorer mental health were more likely to have lost a partner or a child (vs. a more distantly related person), be more recently bereaved, be bereaved due to war-related violent circumstances (vs. other causes of death), have experienced more potentially traumatic events (PTE) in their lifetime, be forcibly displaced (vs. not displaced), and to report higher levels of MI appraisals.*

**Comment:**
7. The authors already explained LPA earlier (unnecessarily), and now they talk about latent profiles again, which is repetitive and conceptually misplaced.

**Response:**
We revised paragraph 5 on pages 4-5 in the introduction and substantially reduced statistical and methodological detail. We retained the reference to profiles in the current study section on page 6 but emphasized their conceptual relevance for interpreting patterns of mental health. Maintaining the term “profiles” in this regard ensures consistency with the Results section and established terminology in the literature.

**Comment:**
8. Methods: The description of sampling channels is very general, and the manuscript does not specify how authenticity of participants was verified (e.g., bots, duplicate responses, non-Ukrainian responders). Online, open recruitment via social media generally requires data quality checks, which are not described.

**Response:**
We clarified the sampling procedures and described the data quality checks implemented in the Methods section to address concerns related to online recruitment.

Page 8:
*Participation was limited to a single entry per individual and restricted to those accessing the survey through a direct Qualtrics link distributed via targeted channels; the survey was not publicly searchable or indexed by search engines. In addition, participants were asked whether they were interested in future research participation and whether they wished to receive study results via newsletters; those who responded affirmatively were invited to provide their email address. In total, 43% (n = 124) participants consented to future contact and 57% (n = 165) requested study results.*

**Comment:**
9. The manuscript states that “written consent was obtained,” which is unclear in the context of an online survey. Please clarify how consent was obtained, documented, and whether the process met ethical requirements for remote data collection.

**Response:**
Before accessing the survey, participants were presented with an online consent form describing the study aims, procedures, voluntary nature of participation, and data protection measures. Participants indicated their consent by actively selecting an agreement option, which was required to proceed to the survey. This procedure complies with ethical requirements for remote data collection and was approved by the local ethics committees.

Page 8:
*Informed consent was obtained digitally before participants accessed the survey.*

**Comment:**
10. Given the highly traumatized and bereaved population, it is essential to report whether mental health resources, crisis hotlines, or debriefing materials were provided. The manuscript does not mention any safeguards, which is a concern.

**Response:**
We thank the reviewer for highlighting the importance of participant safeguards. We have revised the manuscript to clarify the support procedures implemented during the study. After completing the prolonged grief questionnaire, participants received immediate, individualized feedback on their grief levels, categorized into four levels (green, yellow, orange, and red), accompanied by brief descriptions of potential symptoms. Participants in the highest category were explicitly encouraged to seek professional support, such as contacting their general practitioner. In addition, all participants were referred to an external grief-support website available in Ukrainian. These measures were implemented to provide timely support and appropriate guidance for participants who may have experienced distress.

Page 8:
*After completing the PG symptoms measure, participants received immediate feedback on their grief levels, categorized into four levels (green, yellow, orange, and red), each accompanied by a brief description of potential symptoms. Participants in the highest category were encouraged to seek professional support, such as consulting their general practitioner, and all participants were provided with a link to a Ukrainian website we developed that presents psychoeducation about grief (www.Вимірюваннягоря.com). This feedback was intended to provide informational support.*

**Comment:**
11. More information is needed on how fraudulent responses were prevented and how eligibility was confirmed, particularly given open recruitment in public online groups.

**Response:**
We thank the reviewer for raising this important point. Several steps were taken to reduce the likelihood of fraudulent or inauthentic responses. Participation was limited to individuals who accessed the survey via a direct Qualtrics link. In addition, participants were asked whether they were interested in future research participation and whether they wish to receive study results via newsletter; those who responded affirmatively were invited to provide their email address. In total, 43% (n = 124) participants consented to future contact and 57% (n = 165) requested study results. This suggests a level of participant authenticity and engagement. It is also important to note that participants received no compensation for participation, and we have no reason to believe that they would provide false responses by completing the questionnaires more than once and providing faked answers.
We acknowledge, however, that additional verification procedures (e.g., more advanced fraud-detection techniques) were not implemented at the time of data collection. This limitation has now been explicitly addressed in the revised manuscript.

Page 26:
*Finally, although data quality checks were applied, additional procedures to verify eligibility and prevent fraudulent responses (such as attention and consistency checks or geolocation verification) were not implemented and should be considered in future research using online recruitment, particularly in light of the rapidly evolving digital environment.*

**Comment:**
12. Please specify the survey platform

**Response:**
The survey was administered using Qualtrics for data collection.

Page 8:
*Participation was limited to a single entry per individual and restricted to those accessing the survey through a direct Qualtrics link distributed via targeted channels; the survey was not publicly searchable or indexed by search engines.*

**Comment:**
13. The internal consistency of the 6-item PTS scale (α = .75) is relatively modest for a clinical measure and especially for use in LPA, which is sensitive to measurement error. The manuscript would benefit from a more cautious interpretation of this reliability coefficient

**Response:**
We revised the manuscript to adopt a more cautious interpretation of the internal consistency of the PTS scale. Specifically, we avoided strong claims based on this measure and clarified that the reliability coefficient should be interpreted as acceptable but modest.

Page 10:
*In the present study, the internal consistency of the 6-item scale was adequate but modest (Cronbach’s α = .75) and in line with prior research (50).*

**Comment:**
14. The levels of missing data on core indicators (14% for PTS, 31% for well-being) are substantial and may affect the stability and interpretability of the latent profiles. The authors should comment on the potential impact of missing data on their results.

**Response:**
In the revised manuscript, we addressed the potential impact of missing data in both the Results and Limitations sections. Missing data on profile indicators (PG, PTS symptoms, and well-being) were handled using full information maximum likelihood estimation, whereas covariate missing data were handled with multiple imputation. Comparisons between participants with complete and incomplete indicator data showed no differences in sociodemographic or loss-related characteristics, except for time since loss (see Supplementary Material A). The implications of missing data and potential self-selection bias are discussed as limitations warranting caution in interpretation.

Page 11:
*Logistic regression results showed that participants who did vs. did not complete all items measuring PG symptoms, PTS symptoms, and well-being did not differ in terms of sociodemographic and loss characteristics, with the exception of time since loss, such that a longer time since the loss was associated with a higher likelihood of missing data (see Supplementary Material A).*

Pages 26:
*Fourth, missing data on profile indicators (PG symptoms, PTS symptoms, and well-being) were handled using FIML estimation, whereas missing covariate data were addressed using multiple imputation. Although participants with complete and incomplete data did not differ on most sociodemographic or loss-related characteristics (except time since loss), self-selection bias remains a potential limitation.*

**Comment:**
15. Standardization (z-scoring) of scales with very different distributions may distort the indicator structure. The manuscript would benefit from a justification for this decision.

**Response:**
Indicators were z-standardized to place measures on a common scale and to emphasize relative patterns across indicators rather than absolute scores. This rationale is now clarified in the Methods section.

Page 11:
*Indicators were z-standardized prior to analysis to place measures on a common scale and to focus on relative patterns across indicators.*

**Comment:**
16. The sample is overwhelmingly female (95%), limiting generalizability. No discussion of this limitation is provided.

**Response:**
We thank the reviewer for pointing at this; we added a reflection on the gender imbalance to the Limitations section.

Page 25:
*Fifth, the sample was predominantly female (95%), reflecting a common gender imbalance in bereavement research and limiting generalizability to male bereaved individuals. This imbalance may partly reflect the war context, as many men in Ukraine are drafted or volunteering for military service, reducing their availability for study participation.*

**Comment:**
17. Very few participants meet criteria for PTSD, raising questions about including PTS symptoms as core indicators in LPA.

**Response:**
We acknowledge that relatively few participants met criteria for probable PTSD. However, the current study focused on posttraumatic stress symptoms in relation to the loss rather than diagnostic status. Even when not passing clinical thresholds, PTS symptoms may still be present and clinically relevant, particularly in bereaved populations. PTS symptoms are a commonly examined dimension in grief research and frequently co-occur with prolonged grief symptoms. Their inclusion was therefore intended to capture meaningful variation in psychopathology associated with loss, even in the absence of high rates of probable PTSD.

Reviewer #2

**Comment:**
The manuscript, “Profiles of mental health indicators among Ukrainian people recently bereaved in the context of the full-scale Russian invasion,” really hits the mark. It’s timely, carefully put together, and the analysis feels solid. The way it explores grief, trauma, and well-being among people living through war adds real value, and the conclusions fit the data.
Strengths? The study uses validated tools (TGI-SR, PCL-5 short form, MHC-SF, HTQ, MIAS), and the Latent Profile Analysis works well here. Including covariates with a three-step model and handling missing data with multiple imputation shows thoughtful stats. Plus, you’ve got ethical approval, preregistration, and an open-data plan—all good signs of transparency.

**Response:**
We thank Reviewer #2 for their positive and thoughtful evaluation of the manuscript. We appreciate the recognition of the study’s timeliness, methodological rigor, and transparency, as well as the constructive assessment of the analytical approach and conclusions.

**Comment:**
Sampling: About 95% of participants are women, and the sampling wasn’t random. The limits on generalizability need to be spelled out more.

**Response:**
In response, we expanded the Limitations section to more clearly address the implications of gender imbalance for generalizability.

Page 25:
*Fifth, the sample was predominantly female (95%), reflecting a common gender imbalance in bereavement research and limiting generalizability to male bereaved individuals. This imbalance may partly reflect the war context, as many men in Ukraine are drafted or volunteering for military service, reducing their availability for study participation.*

**Comment:**
Missing data: Since the survey had two parts, there’s quite a bit of missing data—up to 31%. FIML and imputation help, but self-selection bias is still a risk and should be mentioned directly.

**Response:**
We expanded the Limitations section to explicitly address the extent of missing data and the potential risk of self-selection bias.

Page 26:
*Fourth, missing data on profile indicators (PG symptoms, PTS symptoms, and well-being) were handled using FIML estimation, whereas missing covariate data were addressed using multiple imputation. Although participants with complete and incomplete data did not differ on most sociodemographic or loss-related characteristics (except time since loss), self-selection bias remains a potential limitation.*

**Comment**:
Interpretation: When you talk about “clinically relevant” cut-offs, make it clear these are probable thresholds, not actual diagnoses.

**Response:**
We have clarified throughout the manuscript that references to clinically relevant cut-offs refer to probable thresholds based on prior research and do not represent formal clinical diagnoses. The revised text now consistently emphasizes that these cut-offs are indicative rather than diagnostic.

Footnote 1: *Throughout the manuscript, “clinically relevant” cut-offs refer to probable thresholds based on prior research and should not be interpreted as formal clinical diagnoses.*

**Comment**:
Also, the idea that PG and well-being are on a single continuum needs a softer touch, since the study’s cross-sectional.

**Response:**
We revised the manuscript to adopt more cautious language when discussing the relationship between prolonged grief and well-being, acknowledging that the cross-sectional design does not allow for conclusions about their dynamic or longitudinal relationship.

Page 22:
*However, given the cross-sectional design, this finding should be interpreted with caution, as relationships between symptomatology and well-being may vary over time.*

**Comment:**
Writing: The manuscript is clear and well written. Still, a few small edits—fixing hyphens, cleaning up spacing, breaking up long sentences—would help it flow better.

**Response:**
We revised the manuscript to address minor stylistic issues, including hyphenation, spacing, and sentence length.

**Comment:**
Data policy: Before publishing, include the final DOI or URL for the planned DANS data upload.

**Response:**
We will include the final DOI for the DANS data repository upon publication and update the manuscript accordingly.

**Comment:**
Bottom line: This is a careful, important study that deserves to be published after a few minor revisions—mainly clarifying sample limits, flagging missing data and possible bias, tempering the theoretical claims, and finalizing data sharing details. The authors really deserve credit for tackling such a crucial humanitarian issue with real care and solid methods.

**Response:**
We thank the reviewer for their careful reading of the manuscript and for their positive assessment of the study’s contribution and methodological rigor. We have addressed the suggested revisions accordingly.
